# Supplementary material for: Development and validation of a prognostic model for mitophagy-related genes in colon adenocarcinoma: A study based on TCGA and GEO databases
Source: PLoS One. 2023 Apr 6;18(4):e0284089. doi: 10.1371/journal.pone.0284089 (PMC10079075; doi:10.1371/journal.pone.0284089)
Supplement: Attachment — Submitted filename: Commendation.docx [file pone.0284089.s002.docx]

In this manuscript, the authors used the bioinformatic methods to predict some mitophagy-related genes for evaluation of survival in colon adenocarcinoma, but it did show several flaws in the design of this study.

1.The language of this manuscript needs to be radically revised.

2.In vitro or in vivo experiments should be conducted to validate the roles of mitophagy-related genes in colon adenocarcinoma.

3.In the GSE17538 dataset, the AUC was lower than 0.65. Therefore, this prognostic model is not reliable.

4.The authors used clinical samples to validate the expression of model genes, which is more convincing and can provide ethical justification?

5. Bioinformatic analysis of the study was too simple. Can authors analyze relation between prognostic models and immunotherapy.
