# Supplementary material for: Development and validation of a prognostic model for mitophagy-related genes in colon adenocarcinoma: A study based on TCGA and GEO databases
Source: PLoS One. 2023 Apr 6;18(4):e0284089. doi: 10.1371/journal.pone.0284089 (PMC10079075; doi:10.1371/journal.pone.0284089)
Supplement: Attachment — Submitted filename: Response to Reviewers.docx [file pone.0284089.s003.docx]

We thank the editors and reviewers for their very helpful comments. Our revision of the reviewers ' comments has greatly improved the quality of the manuscript. The response to the reviewer 's comments is as follows :

Comment:

1.The language of this manuscript needs to be radically revised.

Response:

Thanks for your valuable suggestion. We are very sorry for our negligence.This manuscript language has been modified by a special polishing agency.

Comment:

2.In vitro or in vivo experiments should be conducted to validate the roles of mitophagy-related genes in colon adenocarcinoma.

Response:

Thanks for your valuable suggestion. The western blot experiment has been completed. Due to the novel coronavirus and laboratory conditions, other experiments could not be further completed. The EPHB2 gene in this model has been reported that its high expression reduces the migration and invasion of tumor cells, indicating that the model has a certain significance. ( Senior PV, Zhang BX, Chan ST. Loss of cell-surface receptor EphB2 is important for the growth, migration, and invasiveness of a colon cancer cell line. Int J Colorectal Dis. 2010 Jun ; 25 ( 6 ) : 687-94. doi : 10.1007 / s00384-010-0916-7. Epub 2010 Mar 26. PMID : 20339854. )

Comment:

3.In the GSE17538 dataset, the AUC was lower than 0.65. Therefore, this prognostic model is not reliable.

Response:

Many studies have reported that AUC greater than 0.6 is reliable.(

①. Wang X, Xu K, Liao X, Rao J, Huang K, Gao J, Xu G, Wang D. Construction of a survival nomogram for gastric cancer based on the cancer genome atlas of m6A-related genes. Front Genet. 2022 Aug 5;13:936658. doi: 10.3389/fgene.2022.936658. PMID: 35991573; PMCID: PMC9389082.

②. Yue Q, Zhang Y, Bai J, Duan X, Wang H. Identification of Five N6-Methylandenosine-Related ncRNA Signatures to Predict the Overall Survival of Patients with Gastric Cancer. Dis Markers. 2022 Apr 8;2022:7765900. doi: 10.1155/2022/7765900. PMID: 35774851; PMCID: PMC9239763.

③. Zhang W, Fang D, Li S, Bao X, Jiang L, Sun X. Construction and Validation of a Novel Ferroptosis-Related lncRNA Signature to Predict Prognosis in Colorectal Cancer Patients. Front Genet. 2021 Oct 28;12:709329. doi: 10.3389/fgene.2021.709329. PMID: 34777458; PMCID: PMC8581609.)

Comment:

4.The authors used clinical samples to validate the expression of model genes, which is more convincing and can provide ethical justification?

Response:

The study has passed ethical review ( 2022ER240-1 ) and has uploaded ethical review approvals.

Comment:

5.Bioinformatic analysis of the study was too simple. Can authors analyze relation between prognostic models and immunotherapy.

Response:

Thanks for your valuable suggestion. The relationship between prognostic models and immunotherapy has been analyzed.
