# Supplementary material for: Development and validation of a prognostic model for mitophagy-related genes in colon adenocarcinoma: A study based on TCGA and GEO databases
Source: PLoS One. 2023 Apr 6;18(4):e0284089. doi: 10.1371/journal.pone.0284089 (PMC10079075; doi:10.1371/journal.pone.0284089)
Supplement: Attachment — Submitted filename: Response to Reviewers.docx [file pone.0284089.s004.docx]

We thank the editors and reviewers for their very helpful comments. Our revision of the reviewers ' comments has greatly improved the quality of the manuscript. The response to the reviewer 's comments is as follows :

Comment:

 Most of the questions have been well addressed. However, the progress of immunotherapy need to be systematically summarized before acceptance.

Response:

Thanks for your valuable suggestion. The summary of the progress of immunotherapy has been completed and added to the discussion section.
